# Anti-Hyperglycemic Effects of Bioactive Compounds in the Context of the Prevention of Diet-Related Diseases

**DOI:** 10.3390/foods12193698

**Published:** 2023-10-09

**Authors:** Raz Alfahel, Tomasz Sawicki, Monika Jabłońska, Katarzyna E. Przybyłowicz

**Affiliations:** Department of Human Nutrition, Faculty of Food Sciences, University of Warmia and Mazury in Olsztyn, 45f Słoneczna Street, 10-718 Olsztyn, Poland; razalfahelr@gmail.com (R.A.); tomasz.sawicki@uwm.edu.pl (T.S.); monika.jablonska@uwm.edu.pl (M.J.)

**Keywords:** bioactive compounds, flavonoids, phenolic acids, cardiovascular diseases, obesity, diabetes mellitus, cancer

## Abstract

Diet-related diseases are health conditions primary caused by poor nutrition. These diseases encompass obesity, type 2 diabetes, cardiovascular diseases, osteoporosis, and certain types of cancer. Functional foods and nutraceuticals offer a promising dietary approach to addressing diet-related diseases across various clinical contexts. The bioactive compounds found in these foods are the subject of intensive studies aimed at discovering their anti-hyperglycemic effects, which are beneficial in alleviating chronic diseases and protecting human health. Hyperglycemia is a common risk factor for metabolic disease and mortality worldwide. Chronic hyperglycemic states can lead to many long-term complications, such as retinopathy, neuropathy, kidney disease, heart disease, cancer, and diabetes. This review explores the potential anti-hyperglycemic effects of bioactive compounds, specifically flavonoids and phenolic acids, and their proposed roles in mitigating chronic diseases and promoting human health. By thoroughly examining the existing literature, we investigated the potential anti-hyperglycemic effects of these bioactive compounds and their proposed roles in managing chronic diseases. The goal of this paper was to enhance our comprehension of how these compounds modulate glucose transporters, with the ultimate aim of identifying effective strategies for the prevention and treatment of diet-related diseases. Overall, this review investigated the use of bioactive compounds from functional foods as potential inhibitors of glucose transporters in the context of prevention/treatment of diet-related diseases.

## 1. Introduction

The increasing prevalence of metabolic diseases has become a major concern that has steadily risen over the past two decades, with estimations suggesting that this burden will persistently rise in the forthcoming years. Projections indicate that by 2050, obesity and hypertension are poised to contribute to the largest number of deaths. Therefore, there is an urgent need for effective preventive and therapeutic measures [1]. In Western culture, which is characterized by sedentary lifestyles, the rapid increase in diet-related diseases can be attributed to the widespread availability of highly processed, calorie-dense foods, which are not only easy to consume but also economically favorable [2]. Combined with a lower intake of fruit, vegetables, high-fiber foods, and insufficient physical activity, this might lead to several diet-related diseases, such as obesity, diabetes, heart diseases, stroke, and cancer [3,4]. Nutritional epidemiology, a relatively new field of medical research, investigates the relationship between nutrition and health [5]. Interest in the nutritional field has expanded and continues to grow in relevance to present-day health concerns and diet-related diseases such as cardiovascular diseases, obesity, and diabetes mellitus. Thus, a comprehensive nutrition strategy offers the promise of substantial improvement in diet quality, better health and wellbeing, and lower health care costs [6]. The inclusion of functional foods in our diet may play an important role in preventing and managing chronic diseases due to the containment biologically active ingredients [7].

Bioactive compounds are compounds which have the capability and the ability to interact with one or more component(s) of tissue by presenting a wide range of beneficial effects. They are mainly derived from vegetables and fruits and associated with the prevention of chronic disease through several mechanisms that include a reduction in oxidative stress [8] and the inhibition, enzymatic activation, or modulation of the expression of certain genes [9]. In addition, many of these compounds have been shown to exert anti-proliferative [10], anti-inflammatory [11], and antiobesity [12] effects.

Glucose serves as a vital energy source for mammalian cells and plays a pivotal role as a substrate in protein and lipid synthesis. Facilitated by glucose transporters, its hydrophilic molecule is transported across the cell membrane. Elevated blood glucose levels have been identified as a pivotal trigger for various health complications, including but not limited to diabetes, cardiovascular diseases, and cancer. As hyperglycemia persists over time, it creates an environment that promotes inflammation, oxidative stress, and cellular damage. These deleterious processes, in turn, amplify the risk of chronic diseases. Understanding the intricate web of interactions between hyperglycemia and the molecular pathways involved in chronic diseases is crucial for devising effective preventive and therapeutic strategies. Understanding the link between hyperglycemia and the initiation of chronic diseases is a paramount concern in the context of modern healthcare [13].

Furthermore, it is crucial to acknowledge the evolving field of nutritional epidemiology and its significance in understanding the connection between diet and health. Ongoing scientific research continually reveals fresh insights into how bioactive compounds, commonly found in functional foods, can be employed to alleviate the detrimental effects of hyperglycemia on health. In this context, our review explores the multifaceted potential of bioactive compounds, specifically phenolic antioxidants like resveratrol, quercetin, catechins, and capsaicinoids, with a specific focus on their impact on glucose transporters. By elucidating the complex interaction between these compounds and glucose transporters, our objective is to provide insights into potential strategies for preventing and managing diet-related chronic diseases.

In order to provide a thorough and reliable review of the literature, we employed a rigorous methodology for paper selection. Our approach consisted of a systematic search across reputable academic databases, such as PubMed, Web of Science, and Scopus, using specific keywords related to hyperglycemia, bioactive compounds, glucose transporters, and their effects on chronic diseases. We prioritized peer-reviewed journal articles published within the last decade, focusing on human and animal studies that explored the impact of bioactive compounds on glucose transporter activity and their relevance to the development or management of chronic diseases. We also included systematic reviews and meta-analyses that offered valuable insights into this research area. Our selection criteria excluded studies with unrelated primary objectives, those characterized by inadequate sample sizes or subpar methodological quality, and studies not available in English. Through this systematic approach to paper selection, including strict inclusion and exclusion criteria, we ensured that only the most relevant and high-quality research was included in our review, strengthening the reliability and validity of our findings.

## 2. Diabetes Mellitus

Bioactive compounds, especially numerous polyphenols, play an important role in glucose homeostasis. They exert their effect on carbohydrate metabolism by reducing fasting and postmeal hyperglycemia, enhancing glucose tolerance, and optimizing insulin secretion. In the context of the prevention and treatment of type 2 diabetes, polyphenols offer beneficial effects on the gastrointestinal tract, pancreatic endocrine functions, liver, and insulin-sensitive tissues [14]. The study by Ojelabi’ego et al. [15] discusses the effect of glucose transporters modulation caused by flavonoids contained in red wine and green tea on the prevention of diabetes. Quercetin, epicatechin gallate, and epigallocatechin gallate have been shown to inhibit the glucose transporter GLUT1. The degree of inhibition depends on the interaction of the GLUT1 transporter with these compounds in the outer layer of its sugar-binding site. Moreover, it has been proven that these flavonoids competitively inhibit glucose uptake but are noncompetitive inhibitors of sugar outflow from erythrocytes. The authors of the study indicate that low concentrations of flavonoids act as cis-allosteric activators of glucose uptake, while higher concentrations of these compounds inhibit sugar uptake competitively and inhibit its efflux from cells noncompetitively [15].

Capsaicinoids represent another group of compounds known for their potential antidiabetic effects [16]. A study investigating their hypoglycemic effects was conducted on rats with streptozotocin-induced diabetes. The obtained results demonstrate the beneficial effects of capsaicinoids on various aspects of glucose metabolism. Specifically, after 4 weeks of administration of the capsaicinoid solutions at 3 dose levels—3 (low-dose group, DL), 6 (medium-dose group, DL), and 9 (high-dose group, DH) mg/kg body weight (bw)—insulin levels were significantly increased (by 50.1%, 72%, and 80 5% in DL, DM, and DH groups, respectively) compared with the control group. Moreover, the study revealed improved total sugar content digestibility, with a significant 3.12% decrease in digestibility observed in the DH group compared with the control group. Additionally, food consumption was reduced in both the DM (32.25 ± 1.44 g) and DH (27.25 ± 1.25 g) groups compared with the model group (38.37 ± 1.48 g). Furthermore, blood glucose levels showed a significant reduction in the DM (15.74 ± 4.72 mmol/L) and DH (14.81 ± 3.28 mmol/L) groups compared with the model group (22.95 ± 3.42 mmol/L). The oral glucose tolerance test (OGTT) results also revealed noteworthy differences, with peak blood glucose concentrations occurring after 30 min in the control and model groups, while in the treated groups, the peak occurred after one hour. In the treatment groups, there was a decrease in glycated serum protein and an increase in glycogen concentrations in liver and muscle cells. The addition of capsaicinoids reduces the expression of mRNA and proteins of SGLT1, GLUT2, and GLUT5. These results indicate that capsaicinoids may play a therapeutic role in the prevention/treatment of diabetes [16].

Study by Song et al. [17] showed the inhibitory effect of flavonoids (mainly quercetin) on the GLUT2 transporter, which resulted in a reduced absorption of glucose from the small intestine. It is important to point out that inhibition of GLUT 2 transporter activity was incompetent and reversible. During the study, diabetic rats were given glucose or glucose supplemented with quercetin in various doses. Animals fed glucose alone have been shown to have hyperglycemia of up to 300 mg/dL 30 min after ingestion. On the contrary, when rats received glucose along with a quercetin supplement, a notable decrease in hyperglycemia was observed. Moreover, the effect of quercetin on hyperglycemia was dose-dependent. Data from this study suggested that the most effective dose of quercetin was approximately 60 mg/kg body weight in rats, which is approximately 4 g/kg body weight in humans. Rapid absorption of glucose in healthy people results in rapid fluctuations in blood sugar levels, which can lead to catecholaminergic overactivity. The hypoglycemic properties of quercetin indicate that it is an important food component in the prevention and treatment of diabetes [17]. The validity of the results of this scientific study is confirmed by subsequent research conducted in later years, demonstrating the inhibitory impact of flavonoids, particularly quercetin, on glucose transporters [18,19]. Moreover, obtained data suggest that aglycones of polyphenols inhibit facilitated glucose uptake, whereas glycosides inhibit the active transport of glucose [18].

Transresveratrol and viniferin are two more compounds that influence glucose uptake by porcine enterocytes of the jejunum and ileum. The findings revealed the potential inhibitory properties of these polyphenols on the sodium-dependent glucose transporter SGLT1. Resveratrol exhibited inhibitory effects on glucose uptake in both intestinal segments (mid jejunum 0.44 ± 0.09 nmol/mg protein∙15 s, *p* < 0.05; ileum 0.88 ± 0.11 nmol/mg protein∙15 s, *p* < 0.01), while viniferin resulted in a complete cessation of glucose transport in the membrane vesicles of the ileum and jejunum (statistical analyses could not be measured). When comparing these two compounds, it became clear that viniferin exerted a more potent inhibitory influence on glucose uptake in the small intestine [20].

In the study by Honari et al. [21], the anti-hyperglycemic properties of the hydroalcoholic extract of Thymus caramanicus Jalas (TCJ) shoots were assessed. TCJ contains numerous bioactive compounds such as flavonoids (rutin, quercetin, luteolin) and phenolic acids (rosmarinic and caffeic acids). The study involved diabetic rats that were administered TCJ at doses ranging from 300 to 500 mg per kilogram of body weight, 30 min before a glucose challenge. The results are remarkable, as TCJ demonstrated a significant improvement in oral glucose tolerance test results after 240 min. Furthermore, a 14-day regimen of daily TCJ consumption led to improvements in various parameters. These improvements included increased insulin secretion, favorable alternations in the lipid profile, improved markers of liver and kidney function, and a reduction in the expression levels of sodium–glucose cotransporter 2 (SGLT 2) and glucose transporter GLUT 2 in the kidneys of diabetic rats, with a nearly twofold reduction compared with the control diabetic rats. The results of this study prove the antidiabetic properties of bioactive compounds found in the extract of TCJ, a plant widely used in Iranian folk medicine [21].

A randomized, controlled, double-blind crossover study by Castro-Acosta et al. [22] explored the potential of polyphenol-rich beverages derived from apples and blackcurrants to reduce postprandial blood glucose levels, shedding light on their impact on insulin secretion and glucose-dependent insulinotropic polypeptide (GIP). The study included twenty men (average age 26 years) and five postmenopausal women (average age 57 years) as participants. During the study, volunteers consumed three different types of test drinks on separate occasions, with a minimum 7-day gap between each visit: CON (0 mg polyphenols), AE (1200 mg apple polyphenols), and AE + BE (600 mg apple polyphenols + 600 mg blackcurrant anthocyanins). The findings demonstrated that beverages containing apple polyphenols and those augmented with blackcurrant polyphenols exhibited inhibitory effects on postprandial glycemia and insulin levels, particularly within the initial 30 min following a meal. Notably, the combination of both extracts demonstrated a more pronounced effect. These outcomes were mirrored in the case of GIP. The study further unveiled that polyphenols found in apple extracts, specifically phlorizides and quercetin glycosides, had inhibitory effects on glucose transporters (GLUT2 and SGLT1). Additionally, the interaction between polyphenols showed the potential for beneficial effects, either additive or synergistic, in the context of disease prevention. This synergy significantly contributed to the observed effects on postprandial glycemia and insulin responses, as detailed in the study [22].

In addition to polyphenols, peptides of plant origin also have anti-hyperglycemic properties. The study by Mojica et al. [23] aimed to assess the effect of black bean protein isolate (HPI) and its peptides on glucose absorption. The results indicate that HPI reduced glucose uptake, potentially by blocking glucose transporters and simultaneously reducing the expression of SGLT1 and GLUT2 proteins and their transport from the cytoplasmic stores to the apical membrane. The oral glucose tolerance test in rats showed a 24.5% decrease in postprandial glucose (50 mg HPI/kg body weight). Bean protein fractions, in addition to their hypoglycemic effect, also have antioxidant properties. The above study shows that black bean peptides and proteins can be an inexpensive alternative source of bioactive compounds in food which can be used as an ingredient in blood glucose control in diabetic patients [23].

## 3. Obesity

The global obesity epidemic continues to escalate, affecting an ever-expanding population. At its current trajectory, obesity is poised to become the foremost contributor to poor health, potentially surpassing undernutrition and infectious diseases in its impact. Obesity may be associated with diabetes mellitus, coronary heart disease, certain forms of cancer, and sleep-breathing disorders [24]. In the context of the prevention and treatment of obesity, there are few scientific directions regarding the antiobesity effect of the consumption of bioactive compounds. These effects manifest through a range of mechanisms, including appetite suppression, inhibition of digestive enzymes leading to reduced absorption of lipids and carbohydrates in the gastrointestinal tract, modulation of glucose transporters, stimulation of energy expenditure, mitigation of gut microbiota dysbiosis, amelioration of obesity-related low-grade inflammation, and reduction in oxidative stress [25,26,27].

A systematic review and meta-analysis conducted by John Wong et al. [28] sought to explore the impact of SGLT-2 inhibitor monotherapy on changes in body weight and cardiometabolic profiles. The analysis included randomized controlled trials involving individuals with overweight or obesity but without diabetes. These trials spanned a duration of more than 12 weeks, during which the effects of SGLT-2 inhibitor treatment were compared with a placebo group. The review encompassed a total of eight randomized controlled trials, with a combined cohort of 750 subjects. The results indicate that SGLT-2 monotherapy was linked to a notable reduction in body weight, with an average decrease of 2.32 kg compared with 1.01 kg in the placebo group, representing a mean difference of 1.31 kg. Furthermore, significant reductions in BMI and fasting blood glucose levels were observed. Nevertheless, there were no discernible changes in waist circumference, fat mass, blood pressure, or lipid profile when compared with the placebo group. These findings suggest that SGLT-2 inhibitors hold promise as valuable therapeutic options in the management of obesity. Moreover, sugar transport by GLUT-2, which is overexpressed in pituitary cells and is naturally present in intestinal cells, was inhibited by quercetin [19]. Quercetin, a food ingredient with an excellent pharmacological safety profile, can act as a potent inhibitor of sugar absorption regardless of its transport [19]. Flavonols (one of the groups of polyphenols) show promise as new pharmacological agents in the fight against obesity and overweight.

Turmeric has long been known for its health-promoting properties, with curcumin being one of the primary anti-inflammatory components of turmeric. Scientific evidence supports curcumin’s activity in promoting weight loss and reducing the incidence of obesity-related diseases [29]. The study conducted by Nima Baziar et al. [30] looked at how taking curcumin supplements affects body weight, waist size, and body mass index (BMI). They analyzed eight randomized controlled trials with a total of 520 participants (265 in the curcumin group and 255 in the placebo group). The review found that curcumin supplements led to a significant reduction in BMI and waist circumference, although there was not a notable impact on overall body weight. One possible explanation for these results is that curcumin appears to have a positive effect on reducing visceral fat and abdominal obesity. While direct evidence linking curcumin to the regulation of glucose transporters is limited, it is plausible to hypothesize that curcumin’s anti-inflammatory properties may indirectly influence GLUT expression and function. For example, chronic inflammation is often associated with insulin resistance, which can lead to aberrant GLUT activity. By mitigating inflammation, curcumin may contribute to improved insulin sensitivity and GLUT regulation.

A study conducted by Yuying Wang et al. [31] explored the potential of a combination of cyanidin-3-*O*-glucoside (C3G) and catechin, extracted from black rice and adzuki bean coats, to inhibit pancreatic lipase, a key factor in obesity. The findings demonstrated that when used together, C3G and catechin had a synergistic effect in inhibiting pancreatic lipase. This suggests that they could be valuable for developing dietary supplements or medications to prevent obesity. The results are further corroborated by another study that investigated the inhibitory effects of organic and aqueous extracts from *Hibiscus sabdariffa* on digestive enzymes associated with obesity, such as α-amylase, α-glucosidase, and pancreatic lipase. This study confirmed that phenolic compounds found in *H. sabdariffa* have significant potential in inhibiting these enzymes related to obesity [26].

In a comprehensive meta-analysis conducted by Zhang et al. [27], the effectiveness of dietary polyphenols in managing obesity was assessed by evaluating their impact on various anthropometric measures related to obesity. Specifically, the study considered measurements of waist circumference, hip circumference, and limb circumferences. The analysis aggregated data from 44 articles encompassing 40 randomized clinical trials published between 2010 and 2021. The findings revealed statistically significant reductions in body weight, BMI, and waist circumference when compared with placebo treatments. The observed reductions were approximately 0.36 kg for body weight, 0.13 kg/m^2^ for BMI, and 0.60 cm for waist circumference. Nonetheless, there was no significant effect observed on body fat percentage. The meta-analysis suggested that dietary polyphenols hold promise in preventing and managing obesity among certain adult populations. These results are corroborated by another systematic review and meta-analysis conducted by Akhlaghi M. et al. [32], which similarly indicated the potential of flavonoids in reducing BMI and waist circumference, albeit without a significant impact on body fat mass. It is plausible that the observed improvements in body weight and metabolism are, in part, mediated by the impact of polyphenols on GLUTs. However, direct evidence in this regard remains a subject for future investigation.

## 4. Cancers

Most cancer cells show high glucose metabolism. To obtain a sufficient amount of energy, neoplastic cells increase the expression of glucose transporters, especially GLUT1, which has a high affinity for glucose. Cancer research has well characterized the relation between cancer and altered glucose metabolism. According to the literature data, glucose molecules are mainly oxidized in tumors in order to promote rapid cell division. In contrast, normal cells mainly use oxidative phosphorylation to generate adenosine triphosphate (ATP) in the presence of oxygen. The above-mentioned observation has come to be known as the “Warburg effect” [33]. It is an important stage in carcinogenesis due to the increase in glucose uptake and lactate secretion, which is a consequence of metabolic changes in neoplastic cells [34]. Flavonoids can modulate oncogenic pathways involved in GLUT regulation, thus influencing glucose metabolism in cancer cells.

The study by Gonzalez-Menendez et al. [35] investigated the regulation of the glucose transporters GLUT1 and GLUT4 in androgen-sensitive and -insensitive prostate cancer cells by four different flavonoids (genistein, phloretin, apigenin, and daidzein). These flavonoids have been shown to limit the growth of prostate cancer cells and reduce insulin-dependent and insulin-independent glucose uptake. The most effective in regulating the level of GLUT transporters and glucose uptake were apigenin and phloretin, which is indicated by the antiproliferative effect being the most strongly demonstrated by them [35]. Moreover, apigenin has also been found to reduce glucose transport by limiting GLUT1 at mRNA and protein levels in CD18 and S2-013 pancreatic cancer cell lines [36]. Quercetin and epigallocatechin gallate have been tested for potential anticarcinogenic properties using the samples of breast cancer cells. In a study by Moreira et al. [37], the results show that both polyphenols inhibited glucose uptake by direct and competitive inhibition of GLUT1 and thus suppressed glucose metabolism. Moreover, the ability of these polyphenols to inhibit the activity of the GLUT1 glucose transporter has been confirmed by other studies [38,39].

Another important flavonoid in cancer therapy is hesperetin. In a study by Yang et al. [40], a decreased glucose uptake from the small intestine was observed due to a decrease in GLUT1 expression by hesperetin. Hesperetin has also been found to inhibit insulin-induced glucose uptake by the impaired translocation of glucose transporter 4 (GLUT4) across the plasma membrane. Moreover, the use of hesperetin contributed to the inhibition of phosphorylation of the insulin receptor beta subunit (IR-beta) and a reduction in cell proliferation. Based on the above results, the authors indicate the potential use of hesperetin as an anticancer agent [40]. A study by Azavedo et al. [41] focused on changes in glucose absorption after ingestion of the other polyphenols, including resveratrol, kaempferol, genistein, chrysin, xanthohumol, and myricetin. The study showed that, among the mentioned substances, kaempferol is the most potent inhibitor of glucose analogue uptake, which inhibits the GLUT1 transporter at the mRNA level in breast cancer cells. Moreover, the results of the study indicate the antiproliferative properties of kaempferol.

Resveratrol is another polyphenol that exhibits anticancer properties. It mainly affects the GLUT1 glucose transporter, and its action reduces the level of GLUT1 protein expression, as well as affects the regulation of many transcription factors (such as HIF-1alpha and c-Myc) and signaling pathways (such as AMPK, Wnt, Jnk kinases, sirtuin and histone deacetylase) regulating the expression of this transporter [42]. Gwak et al. [43] showed that resveratrol inhibits glucose uptake in four ovarian cancer cells by disrupting GLUT1 transport across the cell membrane in a manner dependent on the Akt/mTOR pathway. Another study by Jung et al. [44] provided information on the inhibition of glycolysis and glucose uptake in cancer cells induced by resveratrol. This metabolic response was dependent on the ability of resveratrol to block intracellular reactive oxygen species, which then inhibited GLUT1 expression and HIF1α protein accumulation (hypoxia inducible factor 1-alpha) [44]. A study by Cao et al. [45], in turn, proved that resveratrol, by inhibiting the GLUT1 transporter, can significantly reverse the effects of high blood glucose, such as the proliferation, migration, and invasion of pancreatic cancer cells. These results demonstrate the potential of resveratrol in the treatment of pancreatic cancer.

## 5. Cardiovascular Disease

Cardiovascular disease (CVD) is known as the primary cause of death globally, and it is estimated that approximately 17.7 million people die from CVD, accounting for 31% of global deaths. This disease represents an increasing public health problem in many countries [46]. It is worth pointing out that CVD is still a major cause of death in patients with diabetes and that the risk of CVD in patients with T2DM is more than twice that in those without DM [47]. Disorders related to cardiovascular diseases are closely related to hypertension, type 2 diabetes mellitus, hyperglycemia, obesity, and hyperlipidemia and have a multifactorial ethology. A direct relationship between hyperglycemia level and cardiovascular disease morbidity and mortality has been demonstrated. During insulin resistance, several metabolic alterations induce the development of cardiovascular disease. For instance, insulin resistance can induce an imbalance in glucose metabolism that generates chronic hyperglycemia, which in turn triggers oxidative stress and causes an inflammatory response that leads to cell damage [48]. In fact, patients suffering from diabetes and CVD show coronary artery disease, peripheral vascular disease, cerebrovascular disease, diabetic cardiomyopathy, and hypertensive cardiomyopathy [47,49,50,51]. In the context of this disease, one of the main reasons for the increased CV risk was thought to be the deleterious effect of hypoglycemia. Therefore, reducing postprandial hyperglycemia by delaying the absorption of glucose in the body through the inhibition of α-amylase and α-glucosidase is an important approach for treating CVD. Recent reports from large CV outcome trials have proven the positive CV effects of SGLT-2 inhibitors and glucagon-like peptide-1 receptor agonists (GLP-1RAs) in patients with high CVD risk [47,52,53]. The enzyme inhibitors used for this action suppress the digestion of carbohydrates and consequently slow the postprandial plasma glucose rise, thereby delaying the rate of glucose absorption [54].

Many studies reveal the potential role of flavonoids and indicate the hypoglycemic actions of flavonoids in different experimental models and treatments [46,47]. In particular, phytochemicals with antioxidant potential are well-reported to play role in reducing the consequences of oxidative stress in disease development and the aging process, and thus contribute to the overall health-protective effects of foods, particularly fruits and vegetables. In a randomized, placebo-controlled, double-blind trial, it was demonstrated that daily administration of 320 mg of anthocyanins for 24 weeks to diabetic patients lowers fasting plasma glucose and decreases serum levels of LDL cholesterol and triglycerides [55]. A one-year treatment with genistein at a daily dose of 54 mg of Caucasian postmenopausal women with metabolic syndrome resulted in a decrease in fasting glucose, fasting insulin, insulin resistance, total cholesterol, LDL-C, triglycerides, visfatin, and homocysteine and an increase in HDL-c and adiponectin [56]. Furthermore, the administration of 27 g/day of flavonoid-enriched chocolate (containing 850 mg flavan-3-ols (90 mg epicatechin) and 100 mg isoflavones) to patients with type 2 diabetes, for one year, reduced peripheral insulin resistance, improved insulin sensitivity, and led to a decrease in total cholesterol to HDL-c ratio and a decrease in LDL-c [57]. The growing interest in natural products to promote human health has resulted in seaweeds becoming popular due to their high bioactive compound contents, especially those exhibiting effects related to cardiovascular protection and metabolism regulation [53]. Increasing evidence has indicated that increasing the consumption of seaweeds helps decrease the risks of CVD and other related diseases by modulating different biological signaling pathways in vivo and in vitro. Yang et al. demonstrated that oral administration of ishophloroglucin A (IPA) and oxtaphlorethol A (OPA) isolated from I. okamurae and I. foliacea significantly ameliorated glucose intolerance and the fasting glucose levels in high-fat diet (HFD)-fed mice, thereby reducing fasting and 2 h blood glucose levels, as well as stimulated GLUT4 in HFD mouse muscle [53,58,59]. However, a lot of efforts are still necessary to better explore the many pathways of both cardiovascular diseases and diabetes.

## 6. Summary

The bioactive compounds discussed in this review may have significant and clinically relevant effects in lowering blood sugar levels in various clinical conditions. When designing future studies, the risk of potential bias due to other confounding factors such as different diet, lifestyle, and genetic factors should be taken into account. Additionally, factors such as dosages, sources, intervention duration, and frequency of consumption should be taken into account. Understanding the precise mechanisms of anti-hyperglycemic action, bioavailability, and the most effective dosage of these compounds may be useful information for (1) plant breeders seeking to develop new varieties with high levels of these compounds through conventional breeding techniques combined with emerging innovative molecular biology techniques, (2) primary processors aiming to optimize compound levels through pre- and postharvesting strategies, and (3) food technologists looking to increase the levels of these compounds in products using processing technologies to deliver the required amounts in appropriate portions. The information contained in this review is part of the trend of searching for dietary therapies that enable the inclusion of products, nutraceuticals, and functional foods that help prevent or control chronic diseases and their complications. Better quality trials (RCTs) are needed to bring these molecules into mainstream clinical applications at the prevention and treatment level. Further interventional studies are needed to determine the clinical value of supplementation and to identify potential drug interactions between these bioactive molecules and standard anti-hyperglycemic drugs, thus improving their safety [13]. The information presented in this review is summarized in Table 1.

## 7. Conclusions

All bioactive compounds discussed in this review have shown strong evidence of glucose-lowering effects in various clinical conditions of the human body. It seems necessary to create dietary recommendations that take into account the intake of bioactive ingredients in the prevention/treatment of chronic diseases. There is still a need to conduct clinical trials to expand our knowledge of the basic mechanisms of their action, doses, and bioavailability in the human body.

## Figures and Tables

**Table 1:** Anti-hyperglycemic effects of bioactive compounds in the prevention of diabetes mellitus, obesity, cancers, and cardiovascular disease.

Diseases	Bioactive Compounds	Effects	Mechanism of Action	References
Diabetes mellitus	Quercetin	Reduction in fasting and postprandial hyperglycemia, improved glucose tolerance, optimization of insulin secretion	Inhibition of GLUT1 and GLUT2	[15,17]
	Capsaicinoids	Improved digestibility of total sugar content, reduced food consumption, decreased blood glucose levels, enhanced insulin levels	Reduction in the expression of mRNA and proteins of SGLT1, GLUT2 and GLUT5	[16]
	Resveratrol, viniferine	Inhibition of glucose uptake	Inhibition of SGLT1	[20]
	Thymus caramanicus Jalas	Increased insulin secretion, favorable alternations in the lipid profile, improved markers of liver and kidney function	Reduction in the expression of SGLT2 and GLUT2	[21]
	Apple, blackcurrant	Inhibitory effects on postprandial glycemia, insulin levels, GIP secretion	Inhibition of GLUT2 and SGLT1	[22]
	Black bean protein isolate	Reduction in glucose uptake	Reduction in the expression of SGLT1 and GLUT2	[23]
Obesity	SGLT-2 inhibitor monotherapy	Reduction in body weight, BMI, fasting blood glucose levels	Inhibition of SGLT2	[28]
	Curcumin	Significant reduction in BMI and waist circumference, positive impact on visceral fat and abdominal obesity	Potential modulation of glucose transporters	[29,30]
	Cyanidin-3-*O*-glucoside (C3G) and catechin	Synergistic inhibition of pancreatic lipase	Modulation of digestive enzymes associated with obesity	[31]
	Extracts from Hibiscus sabdariffa	Inhibition of α-amylase, α-glucosidase, pancreatic lipase		[26]
	Dietary polyphenols	Reductions in body weight, BMI, waist circumference	Potential modulation of glucose transporters	[27,32]
Cancers	genistein, quercetin, apigenin, daidzein	Inhibition of glucose uptake	Inhibition of GLUT1 and GLUT4	[35,36,37,38,39]
	Hesperetin	Decreased glucose uptake	Inhibition of GLUT1 and GLUT4	[40]
	Kaempferol	Inhibition of glucose analogue uptake	Inhibition of GLUT1	[41]
	Resveratrol	Inhibition of glucose uptake, effects on Akt/mTOR pathway	Inhibition of GLUT1	[41,42,43,44,45]
Cardiovascular diseases	Anthocyanins	Reduction in fasting plasma glucose and lowering of serum levels of LDL cholesterol and triglycerides	Potential modulation of glucose transporters	[55]
	Genistein	Reduction in fasting glucose, fasting insulin, insulin resistance, total cholesterol, LDL-C, triglycerides, visfatin, homocysteine, along with an increase in HDL-C and adiponectin		[56]
	flavonoid-enriched chocolate	Reduction in peripheral insulin resistance, enhancement of insulin sensitivity, decrease in the total cholesterol to HDL-C ratio, as well as a decrease in LDL-C		[57]
	ishophloroglucin A, oxtaphlorethol A	Improvement in glucose intolerance and fasting glucose	Stimulation of GLUT4	[53,58,59]

## Data Availability

No new data were created or analyzed in this study. Data sharing is not applicable to this article.
